# Retinopathy of prematurity: it is time to take swift action

**Published:** 2018

**Authors:** Subhadra Jalali, Rajvardhan Azad

**Affiliations:** Deputy Director: Newborn Eye Health Alliance (NEHA) and Director, Quality: LV Prasad Eye Institute, Hyderabad, India.; Chairman: National Task Force on ROP, Ministry of Health and Family welfare, Government of India and Chairman: Raj Retina and Eyecare Centre, Patna, India.

**Figure:**
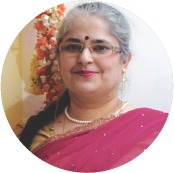
Subhadra Jalali

**Figure:**
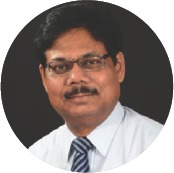
Rajvardhan Azad

**In the 21st century, South Asia faces an additional new challenge of childhood blindness from retinopathy of prematurity (ROP). The epidemic of ROP blindness is here and is rapidly spreading across all countries in South Asia.**

**Figure:**
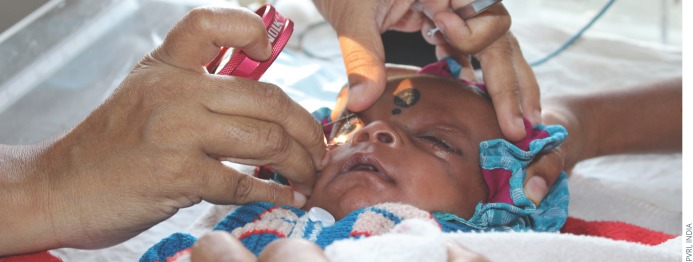
A preterm under going ROP follow up. INDIA

Advances in science and technology, coupled with human compassion, focused goals and enhanced global collaboration, have greatly improved our ability to eliminate some of the most dangerous diseases around the world. Even without the power of the internet, fast transport and communication systems available today, health workers and governments worked together in successfully wiping away the scourge of small pox from the world in the early seventies. The more recent success like prevention of polio and maternal tetanus from large parts of the world encourage many health workers and policy makers to believe that “yes, we can!”

Blindness and vision loss in babies and infants is often not perceived by parents and public as being preventable. Although most health workers are aware of preventable conditions like ophthalmia neonatorum or treatable conditions like congenital cataract, the general public perception remains that babies are born blind, and very little can be done in terms of public health preventive measures. Blindness and eye diseases in young children are considered difficult to diagnose and treat and lack of public awareness of these eye diseases including the absence of formal curriculum in medical or nursing schools, are few of the key challenges in the region. In many places in South Asia, health workers including doctors are not trained to conduct eye screening of newborns along with their systemic health evaluation. It is not uncommon to see children in schools for the blind who have never been screened or examined comprehensively by a trained eye specialist. The biggest assumption in many cases is that the numbers are too small to require the attention of policy makers, health media and blindness prevention programmes. A notable exception in preventing childhood blindness was the successful global Vitamin A prophylaxis programme in India. It was the first of it's kind in which the health workers were trained in identifying ocular signs and symptoms in children. Apart from this initiative, in the absence of any newborn eye screening programmes, largest number of children in South Asia are already facing needless blindness.

In the 21st century, South Asia faces an additional new challenge of childhood blindness from retinopathy of prematurity (ROP). The epidemic of ROP and ROP blindness is here and is rapidly spreading across all countries in South Asia.^1^ What started in a few major cities of South Asia in the late 90's, has now rapidly spread across smaller towns and even rural areas.^2–5^ This is a consequence of rapid scaling up of sick newborn and neonatal intensive care units (SNCU/NICU) to reduce the unacceptably high neonatal mortality rate in South Asia. The association between institutional survival of preterm babies and ROP blindness is well documented in the scientific literature, from the first case recorded in Boston (1942) to the epidemic in middle-income countries.^6^

In the South Asia region almost 5 million babies are born preterm every year, with an estimated 80,000 surviving neonatal care and at risk of ROP. All these babies need to be screened for retinal disease within thirty days of birth to identify those who develop sight-threatening ROP, followed by timely treatment. However, there is often lack of awareness about this emerging condition coupled with challenges of accessibility, affordability, lack of knowledge, inadequately skilled human resource and resource allocation. What is required of us is to establish and expand ROP programmes and build capacities at a very rapid rate - the babies cannot wait, as they become blind within the first few months of birth. Many individuals and child care/eye care teams have evolved different models of ROP care across South Asia that are geared up to meet this challenge.^5^,^7^,^8^ Human resource training, improved quality of neonatal care, fundoscopy screening for the sight-threatening stages of ROP through regular visits by ophthalmologists or telemedicine, capacity building by providing lasers, data registries, curriculum upgrades, scientific publications and numerous medical education presentations are some of the initiatives taken up across the region. Awareness and advocacy have slowly convinced some governments to start considering ROP as a serious and preventable cause of blindness. While substantial work in some regions of South Asia is ongoing, with many success stories, a huge amount of work still needs to be done before this epidemic can be overcome. Combined and coordinated efforts in the South Asian region may help us achieve this goal faster.

**Figure:**
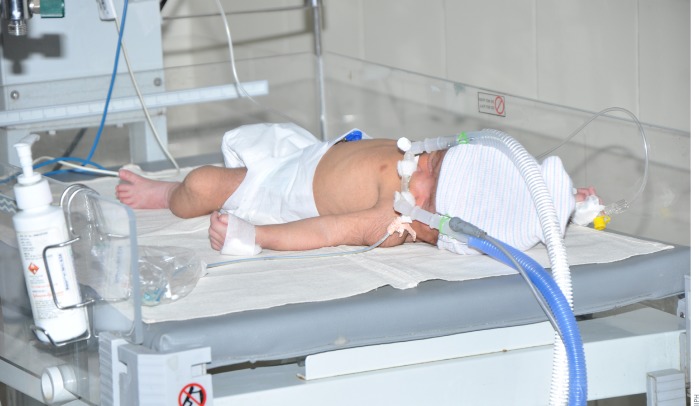
A preterm infant admitted to an SNCU in India.

This issue of the Community Eye Health Journal South Asia brings together the knowledge and experiences of ophthalmologists, neonatologists, paediatricians, care givers, nurses and health workers in an accessible style. Since the region has diverse cultures and terrains, different health systems and financing mechanisms, with huge gaps in human resources, local ROP leaders have modified the models of care to align with local needs for effective implementation. We hope that the experiences documented in this journal will provide an impetus to implement ROP programmes in all communities that have an SNCU/NICU so that no baby goes needlessly blind. Preterm babies can take heart that ROP service providers are all very enthusiastic and working towards their Right to Sight!
